# Viruses as Sole Causative Agents of Severe Acute Respiratory Tract Infections in Children

**DOI:** 10.1371/journal.pone.0150776

**Published:** 2016-03-10

**Authors:** Fleur M. Moesker, Jeroen J. A. van Kampen, Annemarie M. C. van Rossum, Matthijs de Hoog, Marion P. G. Koopmans, Albert D. M. E. Osterhaus, Pieter L. A. Fraaij

**Affiliations:** 1 Department of Viroscience, Erasmus MC, Rotterdam, the Netherlands; 2 Department of Paediatrics, Erasmus MC – Sophia, Rotterdam, the Netherlands; 3 Department of Paediatrics, Paediatric Intensive Care Unit, Erasmus MC – Sophia, Rotterdam, the Netherlands; 4 Research Center for Emerging Infections and Zoonoses (RIZ), University of Veterinary Medicine, Hannover, Germany; Kliniken der Stadt Köln gGmbH, GERMANY

## Abstract

**Background:**

Respiratory syncytial virus (RSV) and influenza A viruses are known to cause severe acute respiratory tract infections (SARIs) in children. For other viruses like human rhinoviruses (HRVs) this is less well established. Viral or bacterial co-infections are often considered essential for severe manifestations of these virus infections.

**Objective:**

The study aims at identifying viruses that may cause SARI in children in the absence of viral and bacterial co-infections, at identifying disease characteristics associated with these single virus infections, and at identifying a possible correlation between viral loads and disease severities.

**Study Design:**

Between April 2007 and March 2012, we identified children (<18 year) with or without a medical history, admitted to our paediatric intensive care unit (PICU) with SARI or to the medium care (MC) with an acute respiratory tract infection (ARTI) (controls). Data were extracted from the clinical and laboratory databases of our tertiary care paediatric hospital. Patient specimens were tested for fifteen respiratory viruses with real-time reverse transcriptase PCR assays and we selected patients with a single virus infection only. Typical bacterial co-infections were considered unlikely to have contributed to the PICU or MC admission based on C-reactive protein-levels or bacteriological test results if performed.

**Results:**

We identified 44 patients admitted to PICU with SARI and 40 patients admitted to MC with ARTI. Twelve viruses were associated with SARI, ten of which were also associated with ARTI in the absence of typical bacterial and viral co-infections, with RSV and HRV being the most frequent causes. Viral loads were not different between PICU-SARI patients and MC-ARTI patients.

**Conclusion:**

Both SARI and ARTI may be caused by single viral pathogens in previously healthy children as well as in children with a medical history. No relationship between viral load and disease severity was identified.

## Introduction

Worldwide acute respiratory tract infections (ARTIs) are among the most important causes of morbidity and mortality in children [1,2]. While most ARTIs result in relatively mild and self-limiting disease, sometimes disease progresses to severe acute respiratory tract infections (SARIs). SARIs are characterized by respiratory failure and are an indication for admission to a paediatric intensive care unit (PICU) because of the need of respiratory support. Some viruses such as respiratory syncytial virus (RSV) and influenza viruses have been shown to be the single cause of SARI, but for other viruses like human rhinoviruses (HRVs) and human coronavirus-NL63 (HCoV-NL63) this is under debate [3–5]. It may be that these viruses cause minimal or mild disease only, but in combination with bacterial co-infections, viral co-infections or alternatively, underlying high-risk conditions infection may result into SARI [6–8]. This hypothesis follows observations of detection of viruses in the nasopharynx of children in absence of clinical signs and symptoms [9–12]. Another explanation for the variable association between infection and severe disease could be the extent of infection, as reflected by viral loads. High viral loads may be associated with increased host cell damage and a more profound immune response, but this relationship has not been confirmed consistently [13–15]. To address the possible causative role of virus infections in SARIs, we performed a retrospective study in our hospital, which has implemented routine testing for fifteen respiratory viruses over the study period.

## Materials and Methods

### Patient and sample selection

We conducted a retrospective study analysing data of paediatric patients (age <18 years) with or without a medical history that were admitted to Erasmus MC-Sophia from April 2007 through March 2012. Erasmus MC-Sophia is a tertiary paediatric referral centre. Clinical and virological data were extracted from the clinical and laboratory databases. We collected all qualitative and quantitative diagnostic virological data obtained with real-time reverse transcriptase PCR (RT-PCR) on respiratory tract samples (nasal washing, sputum, throat swab, nose swab, or bronchoalveolar lavage specimen (BAL)) of patients admitted to PICU or medium care (MC). Nasal washings were obtained by infusing 1–2 ml NaCl intranasally. Samples were obtained on clinical indication. Only samples obtained upon admission or within 72 hours after admission were included. Patients could be included more than once if re-admitted with SARI or ARTI, and a sample was obtained > 35 days after the first discharge and tested positive for a different virus. For further analysis these patients (SARI: n = 1; ARTI: n = 1) were considered new patients. The following clinical data was obtained: age, gender, reason for admission, clinical diagnosis, underlying medical condition, specimen used for RT-PCR, C-reactive protein (CRP)-levels, bacteriological test results, types of respiratory support, length of PICU stay and hospital stay, and final outcome. Based on this clinical data, we subsequently grouped patients with SARI or ARTI as primary reason for PICU or MC admission respectively.

Patients were also categorized according to presence or absence of known risk factors for SARI: pulmonary disease, pre-term birth (born before 37 weeks of gestation), anatomical malformations, syndromal disorders, cardiovascular disease, oncology, immunology, neuro-muscular impairment and other disorders including scoliosis.

### Study groups

We defined SARI as a severe acute respiratory tract infection with the potential need for invasive respiratory support and therefore requiring PICU admission. We defined ARTI as acute respiratory tract infection without the need for intensive care admission and we used this group as a control to study severe disease caused by a single virus infection. In the present study we specifically focused on samples obtained from patients admitted to PICU or MC with SARI or ARTI, caused by a single virus infection in the absence of typical bacterial and viral co-infections (PICU-SARI patients and MC-ARTI patients respectively). We further defined respiratory support into supplemental oxygen need (nasal cannula), non-invasive respiratory support (non-rebreathing mask and optiflow), invasive respiratory support with endotracheal intubation (nasopharyngeal tube, trachea tube, trachea-cannula) and extracorporeal membrane oxygenation (ECMO).

The laboratory diagnostic work-up was RT-PCR testing for fifteen respiratory viruses: adenoviruses (ADV), HCoV-NL63, HCoV-OC43 and HCoV-229E, human bocavirus (HBoV), human metapneumovirus (HMPV), influenza A and B viruses, parainfluenza viruses 1–4 (PIV), RSV type A and B and HRV [16]. Samples with a cycle threshold (Ct)-value < 40 were considered positive. Of note Ct-values are inversely correlated to viral load, and only provide semi-quantitative results. For the purpose of this study we combined RSV type A and B results and we excluded patient samples obtained during an emergency department visit, out-patient-clinic visit or from patients admitted to the neonatal intensive care unit. Positive viral test results were traced back to patients admitted to PICU or MC. We compared all patients with a single virus detected and admitted to PICU with patients admitted to PICU with SARI (PICU-SARI patients), and all patients admitted to MC with patients with ARTI admitted to MC (MC-ARTI patients).

### Exclusion of typical bacterial co-infections

We excluded samples from patients with evidence of typical bacterial co-infections upon admission and within 24 hours after admission when: CRP-levels were > 40 mg/L or tested first time > 24 hours after admission and/or in case of positive bacteriological test results. We defined a sputum positive for bacterial infection when > 10 bacteria per ocular field (/OF) were present in gram staining performed on the sputum (direct examination), respiratory pathogenic bacteria were cultured, and/or commensal bacteria were cultured with growth of > 2 on a scale of 4. The quality of sputum was optimal for testing when it contained < 10 epithelial cells/OF (10 x 10 magnification). Sputum samples containing ≥ 10 epithelial cells/OF observed by direct examination, were only considered sputum of good quality when the leucocyte-to-epithelial cell ratio was ≥ 10/OF. Absence of sputum for bacteriological testing was not a reason for exclusion, as sputum could not be obtained from all children. Furthermore, we analysed other bacterial cultures if performed and a positive culture of cerebrospinal fluid, blood or BAL were defined as bacterial co-infection and thus a reason for exclusion from further analysis. Routine testing for atypical bacterial infections caused by *Chlamydophila pneumoniae*, *Mycoplasma pneumoniae* or *Bordetella pertussis* was not performed.

Three investigators (FM, JVK and PF) independently reviewed the combined laboratory and clinical data from all PICU patients, based on the previously defined selection criteria, to decide on inclusion. Disagreement was resolved by consensus.

### Statistical analyses

Data were analysed using SPSS version 20.0 (IBM, SPSS, Chicago, IL, USA) and GraphPad Prism version 6. For continuous data, medians, interquartile ranges (IQR), lower IQR (LIQR) and upper IQR (UIQR) were calculated. To analyse categorical data we used Fisher’s-exact tests or Chi-square tests depending on the sample size. We calculated the number of viruses present in the study groups. To study the relation between disease severity and viral load, we compared the Ct-values in nasal washings in our study groups with Mann-Whitney U tests. We only included groups with more than six children based on the following power analysis: n = 2*(2.8*SD/Mean)^2, with an estimated mean of 25 and SD of 15 based on the number of patients needed to show a difference in Ct-values.

### Ethics

This study was approved by the Medical Ethical Committee of the Erasmus MC, Rotterdam the Netherlands (MEC 2013–221). Informed consent was waived because this was a retrospective case-chart study. Data were stored anonymously and cannot be retraced to individual patients.

## Results

### PICU-SARI patient characteristics

**Fig 1:**
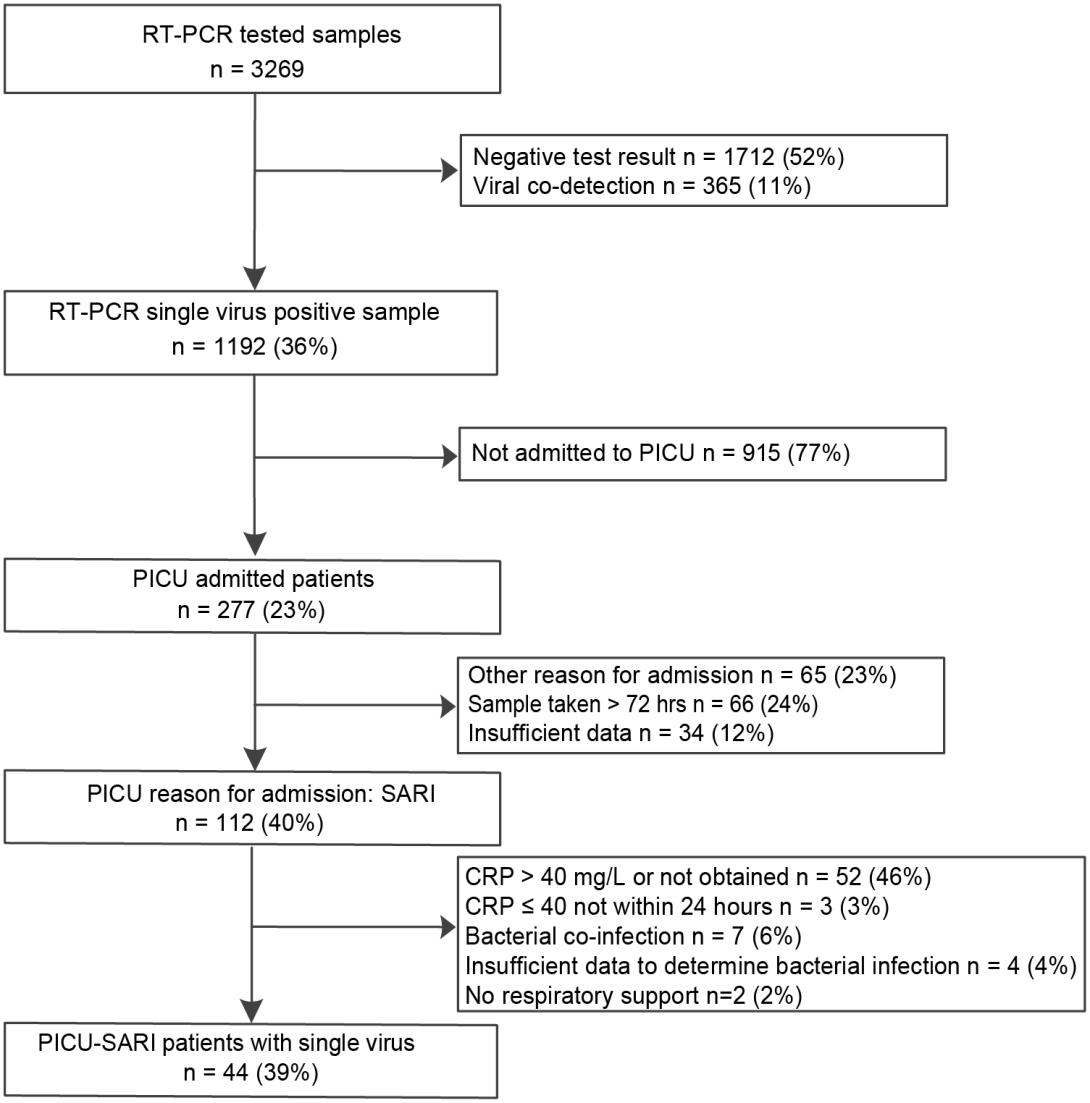
Flowchart patient selection for patients admitted to the paediatric intensive care unit (PICU) with severe acute respiratory tract infections (SARIs) with one respiratory virus, in the absence of viral co-infections and typical bacterial co-infections in children admitted to Erasmus MC-Sophia between 2007 and 2012.

The selection of PICU-SARI patients is outlined in Fig 1 and details of excluded patients are listed in S1 Table. An overview of the epidemiological data is shown in S1 Fig. We identified 44 PICU-SARI patients with a median age of 9.6 months (LIQR 2 -UIQR 25, min 0.46—max 152) and an equal gender distribution (males: 22/44, 50%) (Table 1). One patient was admitted twice because of SARI, with RSV or HRV as single causative agent detected, and with an admission interval of 23.5 months. Thirty-four patients had an underlying medical condition (34/44, 77%). The remaining ten patients were healthy until admission (10/44, 23%) (Table 1). The most common underlying medical condition was pre-term birth (10/34, 29%). Reasons for admission were: obstructive upper respiratory tract infection (URTI) (9/44, 20%), lower respiratory tract infection (LRTI) (15/44, 34%) and severe wheezing with respiratory distress (18/44, 41%). One patient was admitted with an apparent life-threatening event (ALTE (n = 1)) with SARI and one patient with SARI related respiratory fatigue due to muscular disease (n = 1) (2/44, 5%). Respiratory support consisted of supplemental oxygen (9/44, 20%), non–invasive respiratory support including non-rebreathing mask and optiflow was given in eight patients (8/44, 18%), whereas 27 patients required invasive respiratory support (27/44, 61%). For one patient additional ECMO was applied due to insufficient oxygenation (1/44, 2%). RSV was more frequently detected in boys (9/13, 69%), most patients were admitted with bronchiolitis (10/13, 77%), six had no underlying medical condition (6/13, 46%), and eight required invasive respiratory support (8/13, 62%). HRV was detected in six girls (6/11, 55%), most patients were admitted with LRTI (4/11, 36%) or wheezing (4/11, 36%), all patients had an underlying medical condition, and seven patients required invasive respiratory support (7/11, 64%). Overall, the median PICU-stay was 3 days (min 0-max 39), after which patients were either transferred from PICU to MC (n = 14, 32%, after which n = 8 (57%) were transferred to another hospital or discharged n = 6 (43%)), transferred from PICU to another hospitals (n = 22, 50%) or were discharged after PICU admission (n = 8, 18%). Admission duration in other hospitals could not be analysed, as data was not available. The overall hospital stay for patients admitted to both PICU and MC was 7 days (min 3-max 30). Two patients died during PICU admission because of cardiorespiratory failure. One of these was a previously healthy child admitted for cardiorespiratory arrest upon influenza A virus infection, the other was a patient with spinal muscular atrophy (SMA) type-2, who developed respiratory failure due to PIV-3.

**Table 1:** Baseline characteristics of children admitted to the paediatric intensive care unit (PICU) with severe acute respiratory tract infections (SARIs) or medium care (MC) with acute respiratory tract infections (ARTIs) associated with a single virus infection in the absence of viral co-infections and typical bacterial co-infections at Erasmus MC-Sophia from 2007–2012.

	PICU-SARI patients n = 44 (%)	MC-ARTI patients n = 40 (%)	p-values
**Age**			
Months	9.6 (1.8–24.7, 0.46–152)^a^	14.15 (7.6–30.1, 0.7–199)^a^	0.0447
Years	0.8 (0.15–2.1, 0.04–12.7)^a^	1.18 (0.63–2.5, 0.06–16.6)^a^	0.0442
Male	22 (50)	22 (55)	0.66
**Reason for Admission**			
Upper respiratory tract infections	9 (21)	9 (22.5)	
Lower respiratory tract infections	15 (34)	18 (45)	
Wheezing and oxygen need	18 (41)	11 (27.5)	
Others	2 (4)*	2 (5)**	
**Medical history**			
None	10 (23)	6 (15)	0.4 (F)
Pulmonary disease (including cystic fibrosis)	6 (14)	5 (12.5)	ns
Pre-term birth (gestational age < 37 weeks)	10 (23)	4 (10)	0.15 (F)
Anatomical malformations and syndromal	8 (18)	5 (12.5)	0.55 (F)
Cardiovascular	1 (2)	4 (10)	0.19 (F)
Oncology and immunology	-	2 (5)	-
Neuro-muscular, SGA and others	9 (21)	14 (35)	0.15 (F)
**Specimens**			
Nasal washing	34 (77)	39 (98)	0.006 (C)
Sputum	8 (18)	-	
Throat swab	2 (5)	-	
Nasal swab	-	1 (2)	
**Respiratory support**			
Supplemental	9 (20)	30 (75)	
Non-invasive	8 (18)	5 (12.5)	
Invasive	27 (61)	**-**	
Extracorporeal oxygenation	1 (including invasive)	**-**	
None	-	5 (12.5)	
**Length of stay**			
PICU stay days	3 (0–39)^b^	0	0.0002
Hospital stay	0	5 (1–21)^b^	
**Survival**	42 (96)	39 (98)	0.61 (C)

^a^ median, lower interquartile range—upper interquartile range, minimum-maximum

^b^ median, minimum-maximum

* apparent life-threatening event and respiratory fatigue due to muscular disease;

** exacerbation of cystic fibrosis and apparent life-threatening event; F = Fisher-exact-test C = Chi-square test.

### MC-ARTI patient characteristics

**Fig 2:**
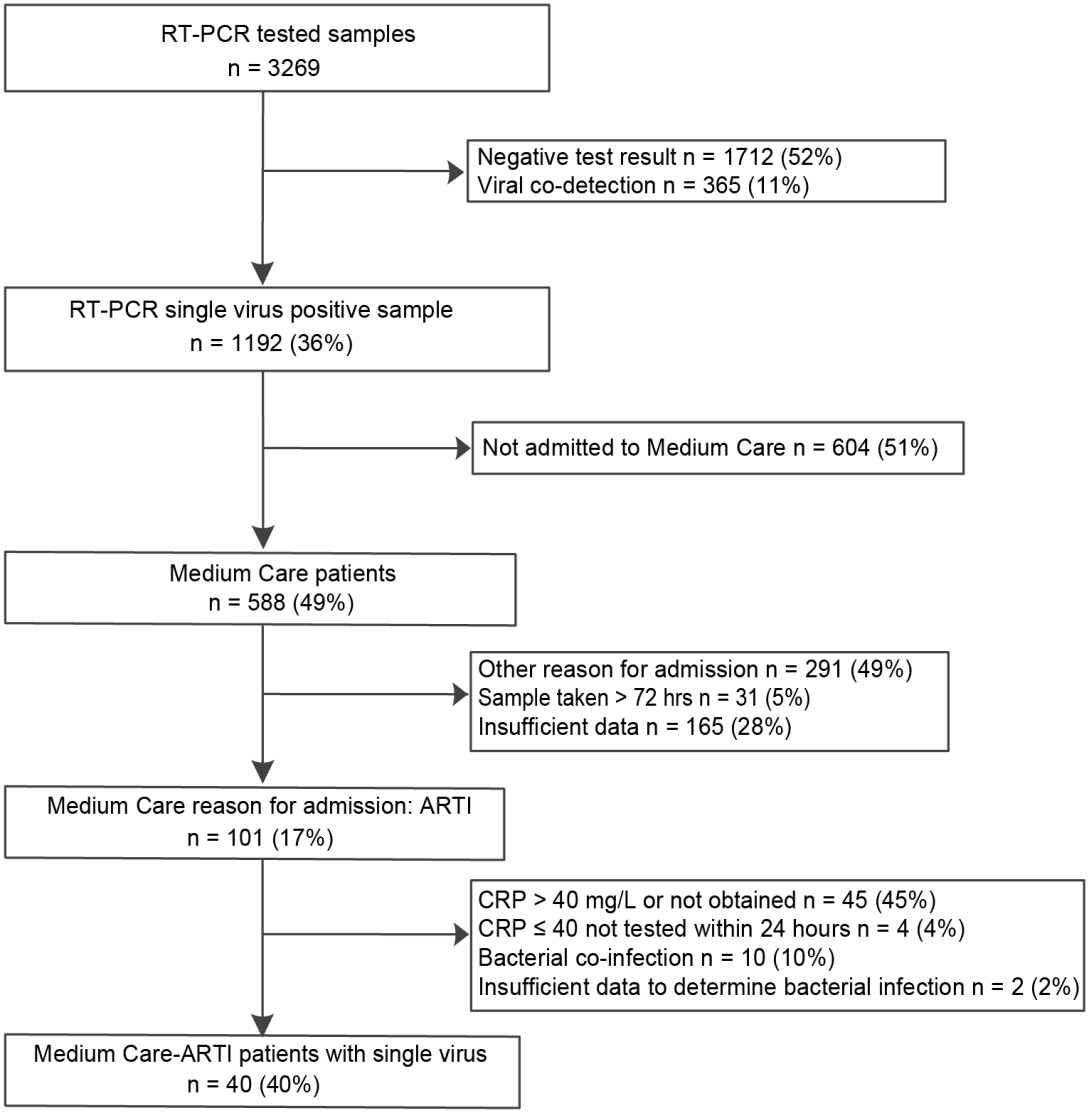
Flowchart patient selection for patients admitted to medium care (MC) with acute respiratory tract infections (ARTI) with one respiratory virus, in the absence of viral co-infections and typical bacterial co-infections in children admitted to Erasmus MC-Sophia between 2007 and 2012.

The selection of MC-ARTI patients is outlined in Fig 2 and details of excluded patients are listed in S2 Table. After using our predefined selection criteria 40 patients admitted to the MC with ARTI were identified. The median age of the MC-ARTI patients was 14 months (LIQR 7.6 -UIQR 30, min 0.7-max 199) with more males than females (22/40, 55%) (Table 1). One patient was admitted twice because of ARTI, with RSV or HMPV as single causative agent detected, and with an admission interval of 1.4 months.

Thirty-four patients had an underlying medical condition (34/40, 85%) including: pulmonary disease (5/34, 15%), a history of pre-term birth (4/34, 12%), and anatomical malformations and syndromal disorders (5/34, 15%) (Table 1). Reasons for admission were: URTI (9/40, 22.5%), LRTI (18/40, 45%) and wheezing (11/40, 27.5%). RSV was most frequently detected in boys (7/13, 54%), most patients were admitted with bronchiolitis (7/13, 54%), ten patients had underlying medical conditions (10/13, 77%), and all RSV infected patients’ required supplemental or non-invasive respiratory support (12/13, 92% and 1/13, 8%). HRV was mostly detected in girls (7/12, 58%), the most common reason for admission was LRTI (7/12, 58%), ten patients had underlying medical conditions (10/12, 83%), and nine required supplemental oxygen (9/12, 75%), one received non-invasive respiratory support (1/12, 8%), and two HRV infected patients did not require respiratory support (2/12, 17%). In addition, one patient was admitted because of an exacerbation of cystic fibrosis with HRV as single pathogen detected (1/12, 8%) and one patient was admitted with an ALTE and ARTI with also a single HRV infection (1/12, 8%). The median hospitalisation stay of MC-ARTI patients was 5 days (1–21 days). In the MC-ARTI group one patient suffering from SMA-type 1 died with an HRV-infection.

### Comparison of PICU-SARI patients with MC-ARTI patients

The median age of PICU-SARI patients was lower than for MC-ARTI patients (9.6 vs 14 months respectively; *p* = 0.045). Specimens tested were mainly nasal washings in both groups (77% and 98%, *p* = 0.006). In addition, CRP-levels were similar between both groups with a median of 6 and 7.5 mg/L respectively (p>0.05). As expected the number of bacterial sputum samples obtained was higher in PICU-SARI patients than in MC-ARTI patients (19/44; 43% vs 8/40; 20%, p = 0.035). Overall, we found 23 patients with a single virus and a bacterial co-infection admitted to the PICU with SARI (S3 Table). Most bacterial co-infections were found in patients with RSV (8/23, 35%) and HRV (7/23, 30%) infections. Eleven patients admitted to the MC with ARTI had a proven bacterial co-infection (S4 Table), and PIV-3 was most frequently co-detected (3/11, 27%). Due to the limited number of proven bacterial co-infections, no associations between detected respiratory virus and bacterial infection could be evaluated. Twenty-three percent of PICU-SARI patients were previously healthy until admission compared to 15% of the MC-ARTI patients (10/44, 23% vs. 6/40, 15%, p = 0.4) (Table 1).

## Detection and Distribution of Respiratory Viruses

Overall in the non selected PICU and MC patients HRV was very often detected and in a lower percentage in samples obtained from PICU patients (31%) than in samples of MC patients (42%) (p = 0.002), whereas RSV (24% versus 15%, p = 0.002) and ADV (10% versus 5%, p = 0.007) were more often detected in samples obtained from PICU patients (Table 2). However, after applying our selection criteria for SARI and ARTI with a single virus only, these differences could no longer be found. Indeed RSV and HRV were represented roughly equally and predominated above all other viruses in both PICU-SARI patients and MC-ARTI patients (RSV: 13/44, 30% and 13/40, 33%, HRV: 11/44, 25% and 12/40, 30%). Besides HRV and RSV nine of the other viruses tested for were associated with SARI and seven of these viruses were also associated with ARTI (except for ADV and HCoV-NL63). Of the ten previously healthy PICU-SARI patients six were infected with RSV (6/10, 60%), three with HBoV (3/10, 30%), and one was infected with influenza A virus (1/10, 10%). Of the six previously healthy MC-ARTI patients, three patients were RSV infected (3/6, 50%), HRV was detected in two patients (2/6, 33%) and one patient tested positive for PIV-1 (1/6, 17%).

**Table 2:** Viral pathogens detected with real time reverse transcriptase PCR in respiratory tract samples of patients admitted to the paediatric intensive care unit (PICU) with or without a severe acute respiratory tact infection (SARI) and medium care (MC) admitted patients with or without an acute respiratory tract infection (ARTI) at the Erasmus MC-Sophia over a 5-year period (2007–2012).

Viruses	PICU-SARI patients with a single virus and no bacterial co-infection n = 44 (%)	All PICU admitted patients with a single virus n = 277 (%)	MC-ARTI patients with a single virus and no bacterial co-infection n = 40 (%)	All MC admitted patients with a single virus n = 588 (%)	p-value between PICU-SARI patients and MC-ARTI patients	p-value between all PICU and all MC patients
**Rhinovirus**	11 (25)	86 (31)	12 (30)	247 (42)	0.6	0.002
**Respiratory syncytial virus**	13 (30)	65 (24)	13 (33)	86 (15)	0.8	0.002
**Adenovirus**	1 (2)	27 (10)	-	28 (5)	-	0.007
**Human bocavirus**	7 (16)	23 (8)	2 (5)	40 (7)	0.2	0.5
**Influenza A virus**	4 (9)	22 (8)	1 (3)	40 (7)	0.4	0.6
**Human metapneumovirus**	1 (2)	12 (4)	3 (8)	23 (4)	0.3	0.9
**Parainfluenzavirus type 1**	2 (5)	10 (4)	1 (2)	9 (2)	ns*	0.08
**Human coronavirus OC43**	1 (2)	10 (4)	2 (5)	16 (3)	0.6	0.5
**Parainfluenzavirus type 3**	1 (2)	7 (3)	5 (13)	36 (6)	0.1	0.03
**Parainfluenzavirus type 4**	1 (2)	6 (2)	1 (3)	13 (2)	ns	ns
**Human coronavirus NL63**	2 (5)	5 (2)	-	20 (3)	-	0.3
**Influenza B virus**	-	2 (1)	-	15 (3)	-	0.1
**Parainfluenza virus type 2**	-	1 (0.4)	-	7 (1)	-	0.5
**Human coronavirus 229E**	-	1 (0.4)	-	8 (1)	-	0.3

* ns, not significant.

### Ct-values of viral pathogens in respiratory tract samples of PICU-SARI patients compared to MC-ARTI patients

**Fig 3:**
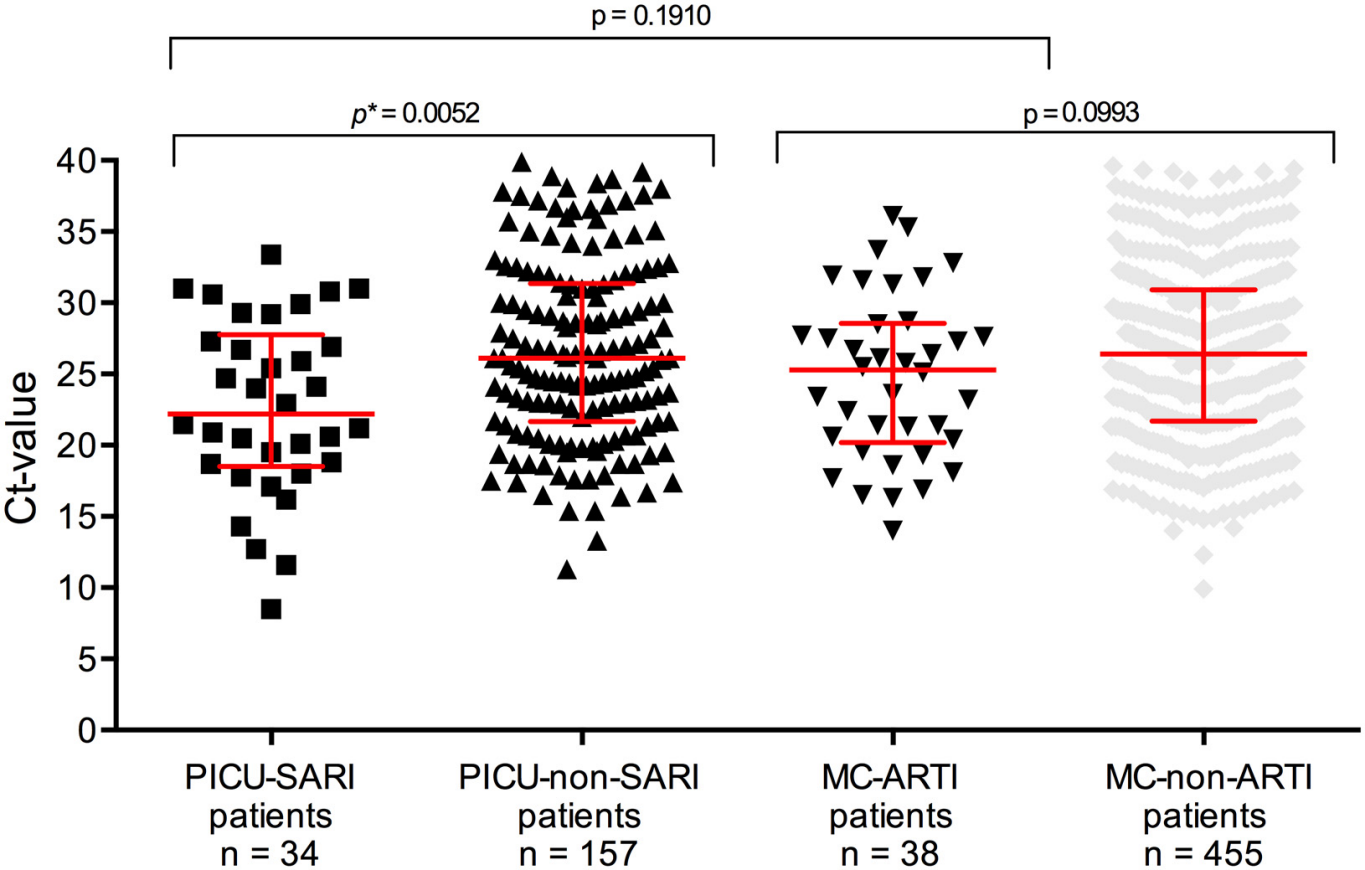
Cycle threshold (Ct) values of all single virus positive samples tested in nasal washings of patients admitted to the paediatric intensive care unit (PICU) with severe acute respiratory tract infections (SARIs) (PICU-SARI patients), all PICU admitted patients, patients admitted to medium care (MC) with acute respiratory tract infections (ARTIs) and all MC admitted patients between 2007 and 2012. (Note: Ct-values were not available for all samples, *with statistical significant difference).

**Fig 4:**
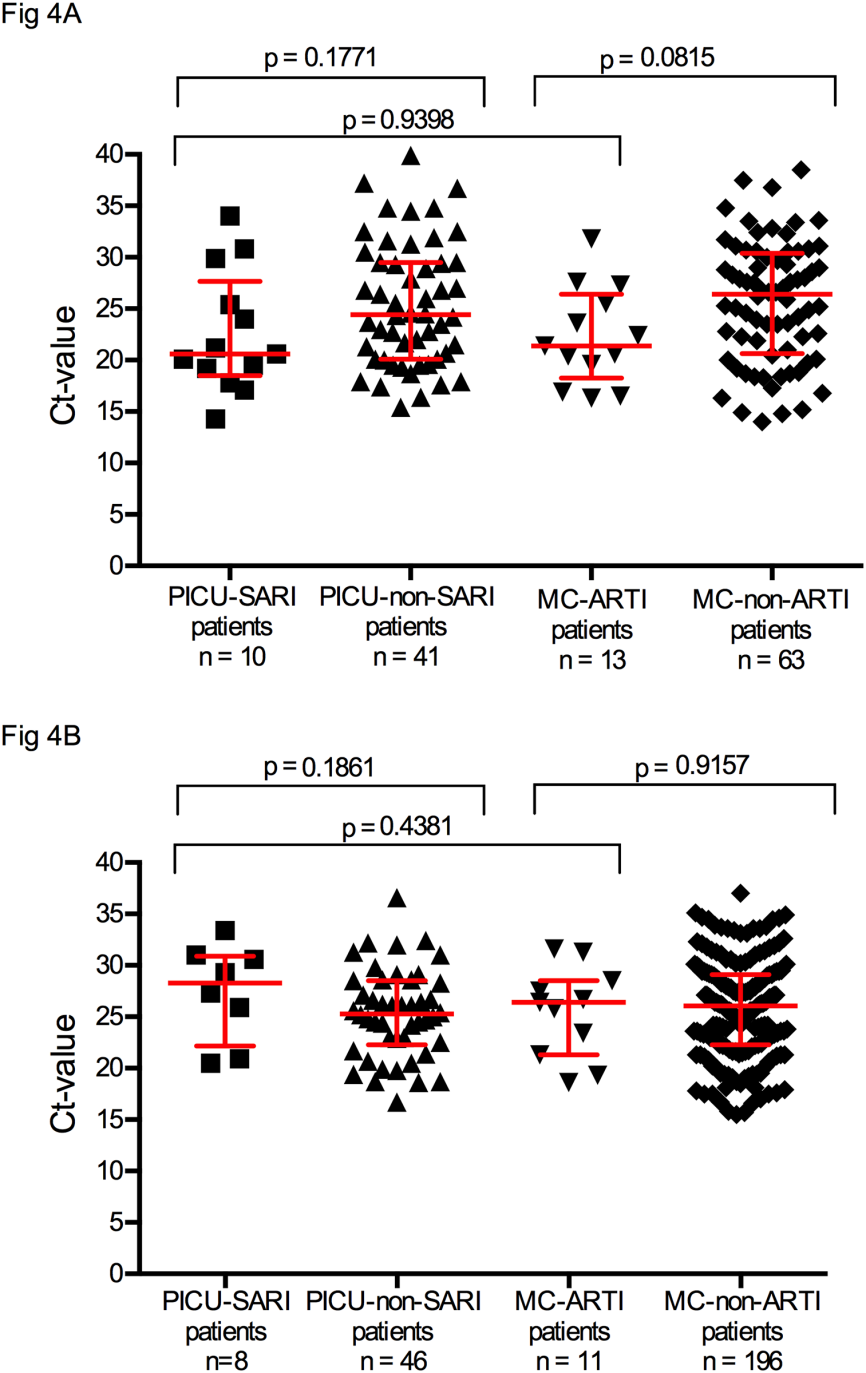
A and B. Cycle threshold (Ct) values of positive samples for respiratory syncytial virus (RSV) (A) and human rhinovirus (HRV) (B) tested in nasal washings and compared between patients admitted to the paediatric intensive care unit (PICU) with severe acute respiratory tract infection (SARI) (PICU-SARI patients), all PICU admitted patients, patients admitted to medium care (MC) with acute respiratory tract infections (ARTIs) (MC-ARTI patients) and all MC admitted patients. (Note: Ct-values were not available for all samples).

In an attempt to relate viral load to disease severity in PICU-SARI and MC-ARTI patients, we compared the total combined Ct-values in nasal washings, which revealed that the median Ct-values were not different between both groups (Ct values 22.2 vs 25.1 respectively; p = 0.191, Fig 3). There was a difference between Ct-values of PICU-SARI patients compared to PICU-non-SARI patients (*p* = 0.005). This was not found for MC-ARTI patients compared to MC-non-ARTI patients (p = 0.0994). We also compared Ct-values of PICU-SARI patients to MC-ARTI patients with and without a medical history and we found no differences between the respective groups (p = 0.289 and p = 0.917, data not shown). Furthermore we compared PICU-SARI patients with invasive respiratory support with PICU-SARI patients and MC-ARTI patients with supplemental and non-invasive respiratory support and we found no difference between these two groups (p = 0.0977, data not shown). Due to limited numbers we were not able to compare Ct-values for all individual viruses identified in this study, but based on our power calculation for both RSV and HRV infected patients, sufficient samples were available to compare Ct-values. No difference could be found in Ct-values between PICU-SARI patients and MC-ARTI patients with median Ct-values for RSV of 20.6 vs 21.4 (p = 0.94) and HRV with 28.3 vs 26.4 (p = 0.44) respectively (Fig 4A and 4B).

## Discussion

In the present paper we have shown that in our tertiary care centre both SARI and ARTI may be caused by single viral pathogens, with RSV and HRV being the most frequent causes. In addition, nine others of the fifteen respiratory viruses tested for were identified as single agents that may be associated with SARI in previously healthy children as well as in children with a medical history. Except for ADV and HCoV-NL63, the remaining seven viruses were also associated with ARTI in previously healthy children as well as in children with a medical history. Our data does not support a relationship between viral load as reflected by surrogate marker Ct-values on the one hand and disease severity reflected by MC or PICU admission on the other. This is counter-intuitive and indeed various studies have indicated that higher viral loads are related to more severe disease as well as locally increased cytokine production [7,14,15,17–25]. Other studies failed to replicate this finding [11,26–29]. Our findings may have been influenced by the selection of hospitalized and seriously ill children. Although Ct-values as presented in our study do not represent an absolute quantitation of viral loads, we did use these semi-quantitative values as a surrogate marker for viral load in our patient samples, as is not unusual in this type of studies [14,20]. Moreover, viral load may be one of many factors that could be associated with disease severity. In this respect the role of a deleterious or counter-productive immune response may be a factor related to disease severity. This has been subject of several studies into the pathogenesis of viral respiratory disease and a better understanding may lead to novel targets for treatment of critically ill patients [30–38].

In accordance with several other studies, RSV and HRV were the most frequently detected respiratory pathogens throughout the study [3,31,39,40]. RSV is a well-established respiratory virus known to cause severe disease by itself, especially in younger children [31,39,40]. For HRV this role as single pathogenic agent has been less well established as it is frequently detected in combination with other respiratory viruses, and has therefore frequently been considered an innocent bystander. We described eleven critically ill patients with HRV as single pathogen detected, supporting other studies that have described HRV as an agent that may cause more or less severe disease [3,41,42]. Less common respiratory viruses such as HCoV and PIV 1–4 were detected, but additional analyses were not performed due to a lack of power.

Previous studies have demonstrated a higher susceptibility to several respiratory viruses in children with certain risk factors such as pre-term birth and broncho-pulmonary dysplasia [39,43]. Indeed pre-term birth was also a marked factor related to hospitalization in our study. In agreement with other studies, RSV proved to be the most common pathogen detected in previously healthy children requiring PICU admission [39,40,44]. It is interesting to note that HBoV and influenza A virus were also found in previously healthy PICU-SARI children. Previously we showed that also HBoV can cause SARI in paediatric patients [25]. Two of our PICU-SARI patients died, which is comparable to previously published frequencies [2,39].

Our selection for a single virus only with an unlikely typical bacterial infection is largely dependent on CRP-levels, which are frequently tested in clinical settings and used to help differentiate between viral and bacterial infections [45,46]. We are aware that the low CRP-levels (≤ 40 mg/L) tested within 24 hours after admission used in our study, may underestimate the occurrence of a bacterial infection or bacterial super-infection. Indeed we cannot fully exclude that some of our patients may have had secondary or pre-existing bacterial infections during hospitalization. We can also not fully exclude the presence of atypical bacteria such as *M*. *pneumoniae*, *C*. *pneumoniae* or *B*. *pertussis* as these were not routinely tested for. Still, considering the very low CRP used to include our patients, we feel that major bacterial involvement was unlikely at the time of hospital admittance [47].

Although this is a retrospective study from one tertiary care centre only, and bacterial cultures could not be performed for all patients for obvious reasons, we identified 44 PICU-SARI patients and 40 MC-ARTI patients infected with one single virus only from a total of 3269 patient samples over a 5-year period. The data generated shed new light on the aetiological role of several respiratory viruses: both SARI and ARTI can be caused by several single viral pathogens in the absence of viral and typical bacterial co-infections.

## Supporting Information

S1 Fig Epidemiology of respiratory virused detected at Erasmus MC-Sophia over a 5-year period (2007–2012). (TIFF)

S1 Table Reasons for paediatric intensive care unit (PICU) admission of non-severe acute respiratory tract infection (SARI) patients (n = 165) with respiratory tract samples tested for fifteen respiratory viruses at Erasmus MC-Sophia over a 5-year period (2007–2012). (PDF)

S2 Table Reasons for medium care (MC) admissions of non-acute respiratory tract infection (ARTI) patients (n = 487) with respiratory tract samples tested for fifteen respiratory viruses at Erasmus MC-Sophia over a 5-year period (2007–2012). (PDF)

S3 Table Bacterial co-infections in single respiratory virus positive patients admitted to the paediatric intensive care unit (PICU) with severe acute respiratory tract infection (SARI) at Erasmus MC-Sophia over a 5-year period (2007–2012). (DOCX)

S4 Table Bacterial co-infections in single respiratory virus positive patients admitted to medium care (MC) with acute respiratory tract infection (ARTI) at Erasmus MC-Sophia over a 5-year period (2007–2012). (DOCX)
