# Supplementary figures and images for: Viruses as Sole Causative Agents of Severe Acute Respiratory Tract Infections in Children
Source: PLoS One. 2016 Mar 10;11(3):e0150776. doi: 10.1371/journal.pone.0150776 (PMC4786225; doi:10.1371/journal.pone.0150776)

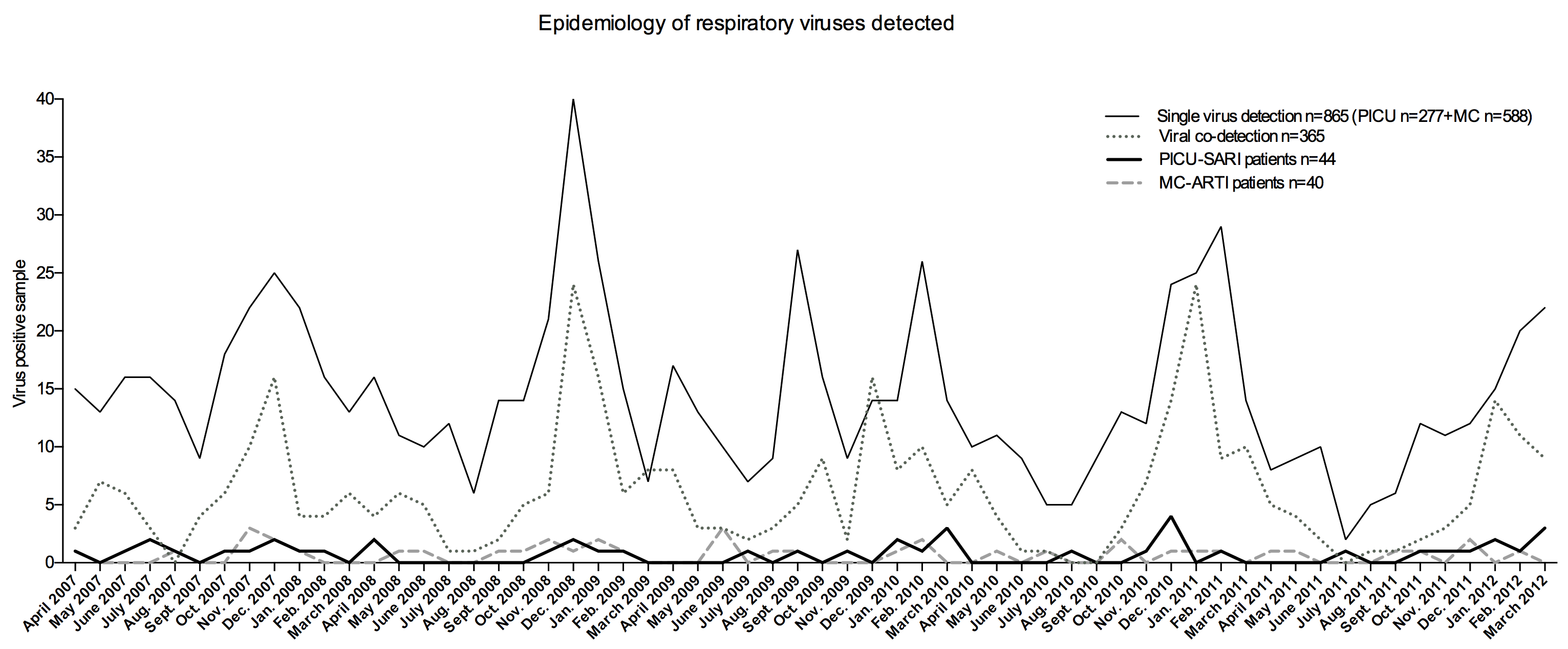

Supplement: S1 Fig — (TIFF) [file pone.0150776.s001.tiff]
