# Supplementary material for: Viruses as Sole Causative Agents of Severe Acute Respiratory Tract Infections in Children
Source: PLoS One. 2016 Mar 10;11(3):e0150776. doi: 10.1371/journal.pone.0150776 (PMC4786225; doi:10.1371/journal.pone.0150776)
Supplement: S1 Table — (PDF) [file pone.0150776.s002.pdf]

S1 Table

Reason for admission PICU-non-SARI patients

**Viruses detected**	**ARTI sample obtained > 72 hours**	**Respiratory non-ARTI**	**Cardio-vascular disease**	**Neurology**	**Gastro-intestinal**	**Anatomical malformations**	**Sepsis like illness / severe systemic infection**	**Neutropenic fever / oncology**	**Others***	**Metabolic disorders**	**Skeletalmuscular disorders**	**Insufficient data available**	Total
**Number of patients**	n=53 (%)	n=13 (%)	n=19 (%)	n=16 (%)	n=7 (%)	n=7 (%)	n=6 (%)	n=3 (%)	n=3 (%)	n=3 (%)	n=1 (%)	n=34 (%)	165
**Rhinovirus**	18 (34)	4 (31)	9 (47)	4 (25)	5 (71)	1 (14)	2 (33)	1 (33)	3 (100)			13 (38)	60
**Respiratory syncytial virus**	12 (23)	4 (31)	2 (11)	2 (13)	1 (14)	4 (57)						5 (15)	30
**Adenovirus**	7 (13)		2 (11)	3 (19)		1 (14)	1 (17)	1 (33)		1 (33)		5 (15)	21
**Human bocavirus**	4 (8)	1 (8)		2 (13)		1 (14)	3 (50)					2 (6)	13
**Influenza A virus**	2 (4)	1 (8)	2 (11)							1 (33)	1 (100)	1 (3)	8
**Human metapneumovirus**				2 (13)				1 (33)				1 (3)	4
**Parainfluenza virus type 1**	3 (6)	2 (15)		1 (6)						1 (33)			7
**Human coronavirus OC43**					1 (14)							1 (3)	7
**Parainfluenza virus type 3**	2 (4)	1 (8)										2 (6)	5
**Parainfluenza virus type 4**	1 (2)		2 (11)									1 (3)	4
**Human coronavirus NL63**	4 (8)		1 (5)	1 (6)								2 (6)	3
**Influenza B virus**				1 (6)									1
**Parainfluenza virus type 2**												1 (3)	1
**Human coronavirus 229E**			1 (5)										1

PICU, paediatric intensive care unit; SARI, severe acute respiratory tract infection; ARTI, acute respiratory tract infection
*Apparent life threatening event (ALTE) in 2 patients, 1 trauma patient
