# Supplementary material for: Viruses as Sole Causative Agents of Severe Acute Respiratory Tract Infections in Children
Source: PLoS One. 2016 Mar 10;11(3):e0150776. doi: 10.1371/journal.pone.0150776 (PMC4786225; doi:10.1371/journal.pone.0150776)
Supplement: S2 Table — (PDF) [file pone.0150776.s003.pdf]

S2 Table

Reason for admission MC-non-ARTI patients

Viruses detected	ARTI sample obtained > 72 hours	Respiratory non-ARTI	Cardio-vascular disease	Neurology	Gastro-intestinal	Urology	Immunology	Skeletalmuscular disorders	Sepsis like illness / severe systemic infection	Neutropenic fever / oncology	Others*	Metabolic disorders	Anatomical malformations	Transfer after PICU admission	Transfer after NICU admission	Insufficient data available	Total
**Number of patients**	n=31 (%)	n=6 (%)	n=41 (%)	n=30 (%)	n=47 (%)	n=16 (%)	n=4 (%)	n=4 (%)	n=18 (%)	n=98 (%)	n=4 (%)	n=6 (%)	n=10 (%)	n=4 (%)	n=3 (%)	n=165 (%)	487
**Rhinovirus**	15 (48)	2 (33)	22 (54)	12 (40)	25 (53)	4 (25)	1 (25)		8 (44)	51 (52)	1 (25)	2 (33)	9 (90)	3 (75)	3 (100)	56 (34)	214
**Respiratory syncytial virus**	3 (10)	2 (33)		2 (7)	7 (15)	1 (6)		3 (75)	2 (11)	12 (12)	1 (25)	2 (33)	1 (10)			24 (15)	60
**Adenovirus**	1 (3)		3 (7)	2 (7)	2 (4)	1 (6)		1 (25)	2 (11)	1 (1)						11 (7)	24
**Human bocavirus**		1 (17)	3 (7)	1 (3)	7 (15)	3 (19)			2 (11)	8 (8)						11 (7)	36
**Influenza A virus**	2 (7)		2 (5)	2 (7)	1 (2)	3 (19)			2 (11)	5 (5)	1 (25)			1 (25)		16 (10)	35
**Human metapneumovirus**	1 (3)		2 (5)	2 (7)						6 (6)						7 (4)	18
**Parainfluenza virus type 1**				1 (3)		1 (6)										5 (3)	7
**Human coronavirus OC43**			1 (2)							2 (2)		1 (17)				6 (4)	10
**Parainfluenza virus type 3**	6 (19)		3 (7)	1 (3)	3 (6)	2 (13)				2 (2)	1 (25)					8 (5)	26
**Parainfluenza virus type 4**	1 (3)	1 (17)	1 (2)	1 (3)						2 (2)		1 (17)				4 (2)	11
**Human coronavirus NL63**			2 (5)	1 (3)	1 (2)		1 (25)		2 (11)	1 (1)						10 (6)	18
**Influenza B virus**			1 (2)	4 (13)		1 (6)	2 (50)			4 (4)						2 (1)	14
**Parainfluenza virus type 2**	1 (3)			1 (3)						1 (1)						4 (2)	7
**Human coronavirus 229E**	1 (3)		1 (2)		1 (2)					3 (3)						1 (1)	7

MC, medium care; ARTI, acute respiratory tract infection

*1 multitrauma patient; 1 patients with suspected child battering; 2 patients admitted for evaluation failure to thrive
