# Supplementary material for: Viruses as Sole Causative Agents of Severe Acute Respiratory Tract Infections in Children
Source: PLoS One. 2016 Mar 10;11(3):e0150776. doi: 10.1371/journal.pone.0150776 (PMC4786225; doi:10.1371/journal.pone.0150776)
Supplement: S3 Table — (DOCX) [file pone.0150776.s004.docx]

**Table S3. Bacterial co-infections in single respiratory virus positive patients admitted to the paediatric intensive care unit (PICU) with severe acute respiratory tract infection (SARI) at Erasmus MC-Sophia over a 5-year period (2007-2012).**

| **Virus detected** | **Ct-value** | **Sputum sample obtained < 48 hours after admission** | **Sputum test result** | **Bloodculture sample obtained < 48 hours after admission** | **Bloodculture test result** | **Other samples tested for bacteria** |
| --- | --- | --- | --- | --- | --- | --- |
| **Respiratory syncytial virus** | 26 | Yes | negative | Yes | negative | NASW^: PCR *M. pneumoniae* |
| **Respiratory syncytial virus** | 20 | Yes | *H. influenzae*, *S. pneumoniae*, *S. aureus* | Yes | negative |  |
| **Respiratory syncytial virus** | 27 | Yes | *H. influenzae*, *S. aureus* | No |  |  |
| **Respiratory syncytial virus** | 22 | Yes | *M. catarrhalis* | No |  |  |
| **Respiratory syncytial virus** | 19 | Yes | *E. coli* | Yes | negative |  |
| **Influenza A virus** | 27 | Yes | > 10 bacteria per ocular field in gram stain | No |  |  |
| **Influenza A virus** | 35 | No (336) | negative | yes | *S. aureus* | BAL**:  *S. aureus* |
| **Rhinovirus** | 18 | Yes | *E. coli* | No |  |  |
| **Rhinovirus** | 24 | Yes | *S. pneumoniae* | Yes | *S. pneumoniae* |  |
| **Rhinovirus** | 22 | Yes | *P. auruginosa*, *Corynebacterium sp.* | No |  |  |
| **Parainfluenza virus type 3** | 33 | Yes | *S. pneumoniae*, *M. catarrhalis* | No | negative |  |
| **Human bocavirus** | 39 | Yes | *K. pneumoniae*, *E.coli* | Yes | negative |  |
| **Adenovirus** | 21 | Yes | *P. auruginosa* | No |  |  |
| **Human metapneumovirus** | 20 | Yes | *S. aureus* | No |  |  |
| **Respiratory syncytial virus** | 24 | Yes | *P. auruginosa*, *S. pyogenes* | No |  |  |
| **Respiratory syncytial virus** | 23 | Yes | *H. influenzae* | Yes | negative |  |
| **Respiratory syncytial virus** | 22 | Yes | *P. auruginosa* | No |  |  |
| **Rhinovirus** | 23 | Yes | *S. pneumoniae*, *S. aureus* | Yes | negative |  |
| **Rhinovirus** | 29 | Yes | *P. aeruginosa*, *M. catarrhalis* | No |  |  |
| **Adenovirus** | 32 | Yes | *P. aeruginosa* | No |  |  |
| **Human metapneumovirus** | 21 | No (72) | negative | Yes | *Coagulase negative S. aureus* |  |
| **Rhinovirus** | 19 | Yes | *M. catarrhalis* |  | No |  |
| **Rhinovirus** | 29 | Yes | *S. aureus* |  | No |  |
