# Supplementary material for: Viruses as Sole Causative Agents of Severe Acute Respiratory Tract Infections in Children
Source: PLoS One. 2016 Mar 10;11(3):e0150776. doi: 10.1371/journal.pone.0150776 (PMC4786225; doi:10.1371/journal.pone.0150776)
Supplement: S4 Table — (DOCX) [file pone.0150776.s005.docx]

**Table S4. Bacterial co-infections in single respiratory virus positive patients admitted to medium care (MC) with acute respiratory tract infection (ARTI) at Erasmus MC-Sophia over a 5-year period (2007-2012).**

| **Virus detected** | **Ct-value** | **Sputum sample obtained < 48 hours after admission** | **Sputum test result** | **Bloodculture sample obtained**  **< 48 hours after admission** | **Bloodculture test result** | **Other samples tested for bacteria** |
| --- | --- | --- | --- | --- | --- | --- |
| **Parainfluenza virus type 3** | 37 | Yes | *P. aeruginosa* | No |  |  |
| **Rhinovirus** | 30 | Yes | *P. aeruginosa* | No |  |  |
| **Parainfluenza virus type 3** | N.A.* | Yes | *S. pneumoniae*, *H. influenzae* | No |  |  |
| **Parainfluenza virus type 3** | 25 | Yes | *S. aureus*, *E. coli* | No |  |  |
| **Human coronavirus OC43** | 28 | Yes | *Aspergillus sp.*, *S. maltophilia*, *P. auruginosa* | No |  |  |
| **Human coronavirus OC43** | 24 | Yes | *M. catarrhalis*, *H. influenzae*, *E.coli* | No |  |  |
| **Respiratory syncytial virus** | 29 | Yes | *S. aureus* | No |  |  |
| **Rhinovirus** | 33 | Yes | *M. catarrhalis* | Yes | negative |  |
| **Influenza A virus** | 32 | Yes | *H. influenzae* | No |  | Throatswab: *C. albicans* |
| **Parainfluenza virus type 4** | 22 | Yes | *P. auruginosa* | No |  |  |
| **Human metapneumovirus** | 37 | Yes | *P. auruginosa* | No |  |  |

*N.A., not available
